# Active case surveillance, passive case surveillance and asymptomatic malaria parasite screening illustrate different age distribution, spatial clustering and seasonality in western Kenya

**DOI:** 10.1186/s12936-015-0551-4

**Published:** 2015-01-28

**Authors:** Guofa Zhou, Yaw A Afrane, Sameer Malla, Andrew K Githeko, Guiyun Yan

**Affiliations:** Program in Public Health, University of California, Irvine, CA92697 USA; Central for Global Health Research, Kenya Medical Research Institute, Kisumu, Kenya

**Keywords:** Active case surveillance, Passive case surveillance, Asymptomatic parasite screening, Transmission hotspot, Age distribution, Seasonality, Temporal changes

## Abstract

**Background:**

Epidemiological characteristics of clinical malaria may differ from asymptomatic infections, thus both cross-sectional parasite screening and longitudinal clinical case surveillance are necessary for malaria transmission monitoring and control.

**Methods:**

In order to monitor malaria transmission, surveillance of clinical malaria from two years of active case surveillance in three cohorts of 6,750 individuals, asymptomatic parasitaemia cases of 5,300 individuals and clinical cases in three study areas were carried out in the western Kenyan highlands in 2009 and 2010. Age distribution, seasonality and spatial clustering were analysed.

**Results:**

The results revealed a significant difference in the age distribution of clinical cases between passive and active case surveillance, and between clinical case rate and asymptomatic parasite rate. The number of reported cases from health facilities significantly underestimated clinical malaria incidence. The increase in asymptomatic parasite prevalence from low to high transmission seasons was significantly higher for infants (<two years) and adults (≥15 years) (500% increase) than that for children (two to 14 years, 65%), but the increase in clinical incidence rates was significantly higher for children (700%) than that for adults (300%). Hotspot of asymptomatic infections remained unchanged over time, whereas new clusters of clinical malaria cases emerged in the uphill areas during the peak season.

**Conclusions:**

Different surveillance methods revealed different characteristics of malaria infections. The new transmission hotspots identified during the peak season with only active case surveillance is an important observation with clear implications in the context of malaria elimination. Both mass parasite screening and active case surveillance are essential for malaria transmission monitoring and control.

**Electronic supplementary material:**

The online version of this article (doi:10.1186/s12936-015-0551-4) contains supplementary material, which is available to authorized users.

## Background

Malaria transmission intensity varies as a result of several factors, including variations in geographic and environmental features, socio-economic factors and the use of preventive measures, such as insecticide-treated bed nets (ITN) and insecticide residual sprays [1-6]. In addition, malaria transmission shows strong age specificity, spatial heterogeneity and changes in seasonal transmission patterns [7-13]. Clinical passive case surveillance (PCS), mass asymptomatic parasite screening (APS) and active case surveillance (ACS) have frequently been used for malaria transmission monitoring and/or intervention effectiveness evaluation [3,4,10,14-19]. However, no attempts have been made to thoroughly compare the differences between these three methods in terms of epidemiological outcomes or the advantages and disadvantages of each method in different transmission settings.

The present study utilizes the community-wide ACS approach alongside PCS and APS to study the age distribution, seasonality and spatial distribution of malaria infections in the highlands of western Kenya. The results will improve the understanding of epidemic mechanisms and improve malaria intervention strategies.

## Methods

### Study site and population

**Figure 1:**
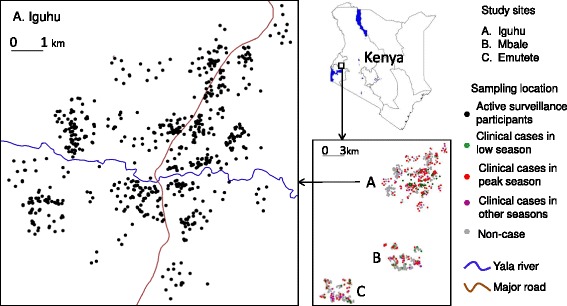
**Study sites and distribution of active case surveillance participants in Iguhu.** Top-right map is the map of Kenya and the study area is marked as a rectangle. Bottom-right map is the locations of the three study sites, A. Iguhu, B. Mbale, and C. Emutete, each dot represents a participated household. Map on the left is the distribution map of active case surveillance participants in Iguhu, each black dot represents a participated household.

The study was carried out in three sentinel sites in the highlands of western Kenya (Figure 1). These sites were: Emutete (34°38′E, 0°03′N) (meso-endemic) in Emuhaya County, Mbale (34°44′E, 0°03′N) (hypo-endemic) in Vihiga County, and Iguhu (34°45′E, 0°10′N) (meso-endemic) in Kakamega County. The catchment population ranged from approximately 23,000 to 28,000 in each of the three study sites. The topography of each of the three study sites is different, but all are characterized by valleys with surrounding hills. Malaria transmission in these sites has been extensively studied [3,4,11,12,20-26]. Four government-run health facilities were selected for the study, and those are the only facilities in the study sites where free malaria treatment was provided and free ITNs were distributed.

### Active case surveillance

At each study site, a cohort of over 1,800 participants was selected randomly from over 20 villages (Table 1) for the longitudinal study. Home locations of the participants were georeferenced with a global positioning system (GPS) handset. Participants’ ages and genders were recorded during the first visit and changes in demography were recorded during subsequent visits. Participants were visited once every other week and screened for clinical malaria. A clinical malaria case was defined as an individual with malaria-related symptoms (fever, i.e., axillary temperature ≥37.5°C, chills, severe malaise, headache, or vomiting) at the time of examination or one to two days prior to the examination, with a *Plasmodium-*positive blood smear. For participants with signs of malaria, blood samples were taken to prepare a thin and a thick smear for parasite species identification [12]. Blood smear tests were conducted in the hospital and confirmed in the Kenya Medical Research Institute (KEMRI) laboratory in Kisumu. Clinical cases were referred to the hospital for free treatment. The cohort was followed from January 2009 to October 2010.

**Table 1:** **Number of individuals/villages participating in different surveillances**

**Surveillance**		**Iguhu**	**Emutete**	**Mbale**	**Total**
Demographic information obtained^§^	Individual	17,190	21,023	18,813	57,026
	No. households	4,803	5,548	4,712	15,063
	No. villages	20	24	22	66
Active case surveillance population^†^	Individuals	1,754	2,320	2,674	6,748
	Female	858	1,302	1,484	3,644
	Cases detected	268	135	74	477
Passive cases surveillance	Cases detected	341	271	690	1,302
Asymptomatic parasite screening population^‡^	Early season	1,898	1,826	1,608	5,332
	Slide positive	90	137	45	272
	Peak season	1,726	1,759	1,798	5,283
	Slide positive	115	200	52	367

^§^These are the base populations for passive case surveillance.

^†^These were the final recruitments and drop-outs were excluded.

^‡^Participants without slide reading results were excluded.

### Passive case surveillance

PCS was conducted at one hospital in Iguhu, one in Mbale, and at two hospitals in Emutete from 2009 to 2010. These were the only government-run health facilities in the study areas where malaria treatment was provided free and free ITNs were distributed. PCS coverage included all people who visited these hospitals. During the survey, patients who were diagnosed clinically with malaria by the clinicians and who agreed to sign a consent and/or assent (for minors under age of 18) form were included in the study. Blood samples were collected by the standard finger-prick method and thick and thin smears were prepared on labelled slides [12]. PCS was conducted in 2009 and 2010. Blood smear tests were conducted in the hospitals and confirmed in the KEMRI laboratory in Kisumu. A clinical malaria case was defined as described in previous section. Only KEMRI-confirmed slide positive cases were considered as clinical malaria.

A demographic database was established for each study site in 2010 (Table 1). The database includes the name, gender and date of birth of each individual participant, as well as the village and GPS location of each participating household. For each PCS case, the name and the village were queried and matched with demographic database records, and the GPS readings were attached for all cases.

### Asymptomatic parasite screening

APS was conducted from February to March and from May to June in both 2009 and 2010. In each of the three study sites, participants were selected randomly from the same area as ACS (Table 1). Upon signing the informed consent/assent (for minors under age of 18) forms, blood samples were collected by the standard finger-prick method [12]. Thin and thick blood smears were prepared for laboratory examination. All slides were examined by two experienced laboratory technicians at KEMRI to identify the parasite species. For quality control purposes, a third technician randomly selected 5% of the slides for re-examination.

### Data analysis

Incidence rates of clinical malaria, for both ACS and PCS, were calculated as ‘the number of cases per 1,000 population per year’. Parasite prevalence of the APS population was calculated as the percentage of positive cases over total examined. Total clinical malaria incidence rates were calculated based on all cases detected via PCS and ACS after removing overlaps between the two methods, and were limited to areas where demographic data were available.

To analyse the differences among different age groups within the same surveillance method and between different surveillance methods, participants were divided into six age groups, i.e., >six months to < two, two to four, five to nine, ten to 14, 15 to 19, and ≥20 years old [3]. Incidence rate was divided into three seasonal groups based on previous studies [12], i.e., early season (January to March), peak season (April to July), which is also the long rainy season in the study area, and the late season (August to December). The χ^2^-test was used to compare the differences in age-specific incidence rates of clinical malaria cases between ACS and PCS, among different sampling seasons, and among different age groups within the same season.

Previous studies have demonstrated that focal clusters along major larval breeding areas exist in asymptomatic parasite infected populations [3,26]. Similar focal clusters may also exist for clinical malaria patients. The null hypothesis is that there is no association between the likelihood of malaria infection rates and the distance from the focal areas of transmission hotspots. In Iguhu, the focal area is the Yala River valley (Figure 1); in both Emutete and Mbale, the focal areas are drainage ditches and streams. In order to evaluate the effects of distance from the focal area based on enrollee demographics, a multivariate logistic regression model was formulated. Malaria infections at different distance intervals were compared and clustering of malaria infections, i.e., significantly low and high infection areas, was identified using stepwise selection of logistic regression. All descriptive and inferential statistical analysis was performed using Statistica 10.0 (StatSoft, Tulsa, OK, USA).

### Scientific and ethical statement

Scientific and ethical clearance was given by the institutional scientific and ethical review boards of the Kenya Medical Research Institute, Kenya, and the University of California, Irvine, USA. Written informed consent/assent (for minors under age of 18) for study participation was obtained from all consenting heads of households and each individual who was willing to participate in the study. Inclusion criteria were: provision of informed consent/assent and no reported chronic or acute illness except malaria. Exclusion criteria were: individuals who were unwilling to participate or infants under the age of six months.

## Results

### Estimation of incidence rate and parasite prevalence

The participating population and detailed characteristics of each survey are shown in Table 1. Demographic information was obtained from 57,026 individuals from 15,063 households in 66 villages or clusters. For ACS, fever was documented in 2,376 ACS visits and clinical malaria was detected in 477 surveillance visits, the annual malaria incidence rate was 40.1 cases per 1,000 population. For PCS, a total of 3,019 probable cases were documented by clinicians and 1,302 clinical cases were detected with blood smears, the annual malaria incidence rate was 20.4 cases per 1,000 population. For APS, a total of 10,615 blood samples were obtained, and parasites were detected in 639 samples. Overall, the asymptomatic parasite prevalence rate was 8.2%.

### Difference in age distribution

**Figure 2:**
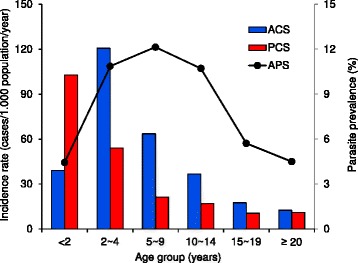
**Age distribution of clinical malaria case rates.** (Pooled 2009 and 2010) detected through active case surveillance (blue bar) and passive case surveillance (red bar) and parasite prevalence detected via cross-sectional parasite screening (black curve).

Due to the limited numbers of clinical incidences, pooled data were used for age-specific incidence analysis. There were significant differences in age distribution and peak age groups among the three surveillance methods (Figure 2, *χ*^2^-test, *P* <0.05). For ACS, the age group two to four years had significantly higher case rates (120.6 cases/1,000 population/year) than any other age groups, and age group five to nine years had the second highest case rate (63.6) (*χ*^2^-test and *P* <0.05); age groups < two years and ten to 14 years had similar case rates (39.1 *vs* 36.6) and difference in case rates was also insignificant between age groups 15–19 years (17.5) and 20 or above (12.5) (*χ*^2^-test, *P* >0.05) (Figure 2). For PCS, the case rates decreased exclusively with increased age (Figure 2), data comparison revealed that the case rate of the age group six months to four years old was significantly higher than that in the rest of the population, and case rates were significantly different among all age groups except between the two oldest age groups (*χ*^2^-test, *P* <0.05). For APS, age groups of two to 14 years had similar parasite prevalence rates (*χ*^2^-test, *P* >0.05) and the rest of the population also had similar parasite prevalence rates (*χ*^2^-test, *P* >0.05), however, the two combined super-groups had a two-fold difference in parasite prevalence (*χ*^2^-test, *P* <0.05) (Figure 2).

### Seasonal changes

In both ACS and PCS, clinical case rates increased about five-fold from early season to peak season (*χ*^2^-test, *P* <0.05) and returned to early season level by late season (*χ*^2^-test, *P* >0.05) (Table 2). Case rates were about two-fold higher in ACS than in PCS in all seasons (Table 2, *χ*^2^-test, *P* <0.05). On the other hand, increase in asymptomatic parasite prevalence from early season to peak season was only about 23%, which was statistically insignificant (Table 2, *χ*^2^ = 1.73, df = 1, *P* = 0.19).

**Table 2:** **Seasonal changes of different surveillance methods**

**Surveillance method** ^**†**^	**Early season**	**Peak season**	**Late season**
ACS (cases/1,000 population/year)	18.9 a	108.4 b	16.0 a
PCS (cases/1,000 population/year)	11.8 a	51.8 b	8.4 c
APS (prevalence rate %)	7.3 a	9.0 b	na

^†^Same (different) letters following the numbers in the same row indicate insignificant (significant) difference tested by *χ* ^2^-test at 5% level. na = not available.

**Figure 3:**
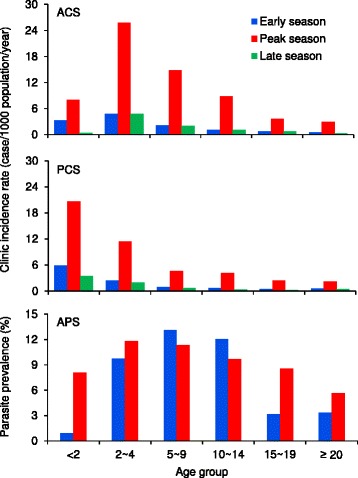
**Seasonal changes in age distribution of clinical malaria incidence rates.** (Pooled 2009 and 2010) in ACS (top) and PCS (middle) and parasite prevalence (bottom) No. APS was done in late season.

Although case rates varied among age groups, the age distribution of the case rates of both PCS and ACS increased significantly in all age groups from the early season to the peak season (Figure 3, *χ*^2^-test, P <0.05), and the magnitude of increase in case rates was similar (three to six-fold) across all age groups in both PCS and ACS (Figure 3, Additional file 1). Case rates dropped to early season level in the late season in all age groups and in both ACS and PCS although magnitude of drops varied (Figure 3, Additional file 1). However, age distribution patterns of case rates in both ACS and PCS remained unchanged over the seasons (Figure 3, *χ*^2^-test, P >0.05), whereas, the age distribution of parasite prevalence changed significantly between seasons (Figure 3, Additional file 1). Differing from clinical malaria, parasite prevalence in children two to 14 years remained almost unchanged from the early season to the peak long rainy season (Figure 3, *χ*^2^-test, P >0.05, Additional file 1). However, parasite prevalence in infants < two years and adults ≥15 years increased significantly from early season to peak season and the increase was more pronounced in infants (nine-fold increase) (Figure 3, *χ*^2^-test, *P* <0.05, Additional file 1).

### Malaria transmission hotspot

**Figure 4:**
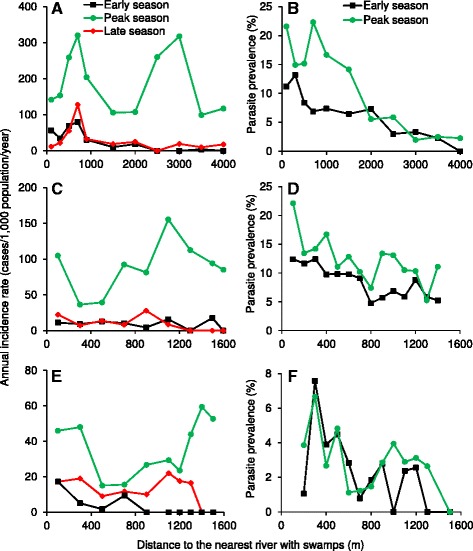
**Graphs of incidence rates.** (Cases/1,000 population/year) (left panel) and parasite prevalence (%) (right panel) against distance to the nearest river with swamps in Iguhu **(A and B)**, Emutete **(C and D)** and Mbale **(E and F)** in western Kenya.

Spatial distribution of clinical cases and asymptomatic infections were very different from each other in all study sites and seasons (Figure 4). Parasite prevalence decreased with increased distance to the nearest river at all study sites regardless of season (Figure 4B, D and F), and an increase in parasite prevalence in peak season occurred mainly close to the river and not in the uphill areas. On the other hand, there was significant increase in incidence rates everywhere in the study area during peak season, and the increase in incidence rates was very pronounced in the uphill area, i.e., high incidence also occurred farther away from the rivers (Figure 4A, C and E).

For asymptomatic parasite infections, after adjusting for the effects of age, the distance to the major breeding habitat is the only factor affecting parasite distribution (Figure 4). For example, in Iguhu (Figure 4B), people living <500 m away from the Yala River valley had twice the possibility of being infected (logistic regression odds ratio *OR* = 2.02, 95% CI (1.37, 2.93)), whereas people living 2,000 m or farther away from the valley had one-fourth the probability of being infected (*OR* = 0.23, 95% CI (0.06, 0.61)). This result is similar to the result published five years ago [3], indicating that the hotspots of asymptomatic infections remained in the same area and did not change between seasons. These results are similar to the findings from the study in the lowland area of coastal Kenya [10].

In contrast, data analysis demonstrated that the spatial distribution of clinical cases detected via ACS had changed between seasons. Clinical cases aggregated around major breeding habitats during the early season of the year, but new incidence clusters formed in the uphill areas during the long rainy season when transmission peaks (Figure 4A, C and E). For example, in Iguhu, during the early season, clinical cases clustered in the area within 800 m of the major river (logistic regression, *OR* = 2.92, 95% CI (1.76, 4.77)), while residents who lived 2,500 m or farther away from the major river valley were eight times less likely (*OR* = 0.12, 95% CI (0.01, 0.56)) to develop clinical malaria during this early period. During the long rainy peak season, clinical malaria clusters still existed in the valley area (*OR* = 1.96, 95% CI (1.33, 2.87)), but new hotspot clusters were detected in the area 2,500-3,000 m away from the Yala River valley (*OR* = 2.80, 95% CI (1.49, 5.14)) (Figure 4A). It is interesting that the case rate in the mid-hill area (1,000-2,000 m from the major river) was significantly lower than that in both the valley and uphill areas during the long rainy season (*OR* = 0.38, 95% CI (0.18, 0.71)). Similar spatial distribution patterns of malaria cases have also been observed in the other study sites (Figure 4C and E).

## Discussion

The three surveillance methods used in this study has their own advantages and disadvantages in estimating malaria burdens. PCS can easily cover a large area but with limited accuracy, ACS can accurately estimate clinical malaria burden but can only be done in relatively small population, APS focuses on asymptomatic infections which is usually the hidden parasite reservoir. The results of this study showed that different surveillance methods reveal different epidemiological profiles. Recent studies show that clinical reports severely underestimate the morbidity and mortality of malaria [27-29]. In this study, PCS detected only about half of the episodes that would have been identified by ACS; this is similar to the findings from previous studies [18,30]. The difference in age structure of clinical cases between PCS and ACS may be biased by the treatment-seeking behaviour. Infants were likely often sent to hospitals for treatment when they are sick, whereas, older children were likely left home when they have mild symptoms. Although PCS provides an underestimate of the burden, since one can efficiently cover a large area with PCS and the variations in PCS match variations in ACS, therefore, PCS may well be sufficient for malaria control programmes with limited resources. APS focuses on the detection of asymptomatic infections, which can be epidemiologically different from symptomatic clinical infections. APS is essential for malaria elimination because asymptomatic infections are hidden reservoir, they are not usually treated and can transmit malaria silently.

The identification of malaria transmission hotspots through active surveillance has been recommended as the key surveillance method to combat malaria [3,8,10,19,31]. Malaria transmission in the highlands was traditionally low and seasonal [12], and transmission was usually focal and attributed to local environmental factors [8]. One surprising finding is the hotspot of clinical malaria cases detected in the uphill area during the high transmission season. This hotspot had not been found during the low season and not in asymptomatic infections. The hotspot clusters along the major breeding habitats can be explained by the frequent exposure to vectors [3,25,32]. The transmission hotspot in the uphill area had not been detected in the past. One possible reason is the surveillance methods and transmission indicators used. Previous studies focused primarily on vector density and asymptomatic parasite prevalence; ACS has not been implemented in the highlands of western Kenya [3,8,32]. The formation of the clinical case hotspots in the uphill area is very likely caused by the lack of adequate protective immunity in the community, because that community was mainly exposed to infections during rainy season when breeding habitats moved up and vector densities build up after the rain [10,25,33,34]. In addition, seasonal streams, which provide temporary breeding habitats, occur in the uphill area during the rainy season. These do not occur in mid-hill where rainwater is adequately drained. The transmission hotspot in the uphill area may explain the infamous outbreak of highland malaria [35-37].

The differences in age distribution between clinical malaria and asymptomatic infections may be explained by the acquired immunity. Young infants are protected by maternal immunity passively transferred across the placenta from mother to foetus, whereas older children and adults have developed essential protective clinical immunity. It is generally believed that this immunity can protect people from severe malaria [38,39]; whether or not this immunity can protect people from developing uncomplicated malaria is unknown. Previous studies also found that school-aged children five to 14 years of age consistently had the lowest ITN coverage [3], thus they have the highest chance of being repeatedly exposed to mosquitoes. These together can at least partially explain the high case rate in young children but high parasite prevalence rate in older children. The drop of clinical cases from peak season to late season is likely due to the decreased vector density, thus decreased exposure to malaria infection after the long-rainy season [12,23,25]. However, it is difficult to explain why clinical malaria increased in everyone from early season to peak season whereas parasite prevalence only increased in infants and adults but not in children two to 14 years. It is speculated that this is the limit of transmission endemicity. As mentioned earlier, due to lack of preventative protection, older children were infected the most among different age groups. This lack of protection occurred all around the year, therefore, infection rates may not change much in this population when season changes. However, the other people were also affected by the seasonal change of exposure. But this needs further studies.

## Conclusion

Different surveillance methods reveal different characteristics of malaria epidemiology. Age distribution and transmission hotspots are different between results obtained by mass asymptomatic parasite screening and by clinical malaria surveillance. Data from government-run health facilities significantly underestimate clinical malaria incidence in rural Kenya; ACS complements this data by monitoring the clinical malaria burden. The new transmission hotspots identified during the peak season only by active case surveillance, which is an important observation with clear implications, this is especially useful in the context of malaria elimination. The results also remind policymakers that although parasite prevalence is the most commonly used indicator of malaria transmission [40,41], decisions on the management of clinical malaria must be made based on epidemiology of clinical malaria.

## Additional file

Additional file 1:
**Fold-changes in incidence rate and parasite prevalence between seasons.** Fold increase from early season to peak season and fold drop from peak season to late season in case rate or parasite prevalence in different age groups by different surveillance methods.
