# Supplementary material for: Active case surveillance, passive case surveillance and asymptomatic malaria parasite screening illustrate different age distribution, spatial clustering and seasonality in western Kenya
Source: Malar J. 2015 Jan 28;14:41. doi: 10.1186/s12936-015-0551-4 (PMC4318448; doi:10.1186/s12936-015-0551-4)
Supplement: Additional file 1: — Fold-changes in incidence rate and parasite prevalence between seasons. Fold increase from early season to peak season and fold drop from peak season to late season in case rate or parasite prevalence in different age groups by different surveillance methods. [file 12936_2015_551_MOESM1_ESM.docx]

Additional File 1.

Title: Fold-changes in incidence rate and parasite prevalence between seasons

Description: Fold increase from early season to peak season and fold drop from peak season to late season in case rate or parasite prevalence in different age groups by different surveillance methods.

| Age group (years) | Fold increase from early season to peak season | | |  | Fold drop from peak season to late season | |
| --- | --- | --- | --- | --- | --- | --- |
|  | ACS | PCS | APS |  | ACS | PCS |
| 0.5~2 | 2.4 | 3.5 | 8.9 |  | 18.3 | 6.0 |
| 2~4 | 5.4 | 4.7 | 1.2† |  | 5.3 | 5.6 |
| 5~9 | 6.9 | 4.7 | 0.9† |  | 7.1 | 6.1 |
| 9~14 | 8.0 | 6.3 | 0.8† |  | 8.0 | 10.6 |
| 15~19 | 5.0 | 5.0 | 2.7 |  | 5.0 | 8.8 |
| ≥20 | 5.0 | 3.7 | 1.7 |  | 11.4 | 5.1 |

† Fold increase/drop was insignificant at level of 5% by χ^2^-test, otherwise they are all significant at level of 5%.
